# Early Detection and Prevention of Ovarian Cancer

**DOI:** 10.3390/cancers17244006

**Published:** 2025-12-16

**Authors:** Lauren Montemorano, Laura Huffman, Lisa Barroilhet

**Affiliations:** 1 Department of Obstetrics and Gynecology, Kaiser Permanente, Oakland, CA 94611, USA; lmontemorano@gmail.com; 2 Carbone Cancer Center, University of Wisconsin, Madison, WI 53792, USA; huffman3@wisc.edu

**Keywords:** ovarian cancer, prevention, early detection

## Abstract

**Background/Objectives**: Epithelial ovarian cancer (EOC) encompasses ovarian, fallopian tube and peritoneal malignancies. It is a deadly disease and is rarely cured when diagnosed at advanced stages. Early-stage disease is often curable, but clinicians and researchers have been stymied in their attempts to reliably screen for this disease, even in high-risk populations. Effective prevention of ovarian cancer is usually limited to the use of combined oral contraceptives and removal of the ovaries and fallopian tubes. **Methods and Results**: We aim to review the current guidelines and the evidence reported for both the early detection and prevention of ovarian cancer. Novel imaging techniques, biomarkers, and surgical advances will be discussed. **Conclusions**: This review will offer (a) an understanding of the epidemiology of EOC (b) analysis and a discussion of relevant molecular markers that might be exploited for more accurate early detection (c) medical and surgical methods to prevent ovarian cancer.

## Simple Summary

Epithelial ovarian cancer (EOC) is the second most fatal gynecologic malignancy, with an estimated 21,410 new cases and 13,770 deaths reported in the United States in 2021. Throughout this paper, the term “ovarian cancer” will refer to malignancies originating in the ovary, fallopian tube, and peritoneal cavity, as these share similar histologic features and are managed using the same treatment strategies. While early-stage ovarian cancer is often curable, the majority of patients—approximately 65%—present with advanced disease. These cases typically require extensive surgery and chemotherapy and are associated with high recurrence and mortality rates. Consequently, improving early detection and implementing effective prevention strategies remain critical priorities. Currently, there is no screening test that has been shown to reduce mortality from ovarian cancer, even in high-risk populations. Prevention strategies are typically limited to surgical intervention. This review article summarizes the current state and potential future directions for the early detection and prevention of ovarian cancer.

## 1. Introduction

Epithelial ovarian cancer (EOC) constitutes the second most lethal gynecologic malignancy, with a projected 21,410 new cases and 13,770 deaths recorded in the United States in 2021 [1]. For the purposes of this chapter, the term “ovarian cancer” will be used to describe cancers arising in the ovary, fallopian tube, and peritoneal cavity, all of which are histologically similar and treated identically. In ovarian cancer, early-stage disease is frequently curable. Unfortunately, the majority (upward of 65%) of patients present with advanced-stage disease, requiring invasive surgery and chemotherapy, with high rates of relapse and death [2]. Early detection and prevention are areas of critical need.

When we consider a patient’s risk of ovarian cancer, the strongest patient-related risk factor is increasing age. The median age at diagnosis is 63 years old. The incidence of ovarian cancer increases with each additional year of life, climbing from 15.7 per 100,000 age 40 years to 54 per 100,000 at age 70 years [3]. Other reproductive factors can alter risk. Pregnancy and parity are associated with a decreased risk of ovarian cancer. Compared to nulliparous women, those who have had one live birth have approximately a 25% decrease in risk of ovarian cancer, while women with two or more live births have an approximate 42% risk reduction. Other protective factors include breastfeeding, bilateral tubal ligation, hysterectomy, and oral contraceptive pills, particularly with more than five years of continuous use [4].

Genetic factors, including family history and germline mutations, can drastically increase a woman’s lifetime risk of developing ovarian cancer. Up to twenty percent of ovarian cancers are related to identified, inheritable mutations in certain genes. Hereditary ovarian cancer syndromes are most often linked to BRCA1 and BRCA2 genes, which are considered the highest risk genes, with lifetime risks as high as 46% and 20%, respectively [5]. Many other genes are emerging as being associated with higher risk, including BRIP1, PALB2, RAD51C, RAD51D, and SMARCA4, [6] with increased risks between 5–15%. Other patient-related factors that are associated with higher risk of ovarian cancer include race (white women are at highest risk), early menarche and later menopause [7].

## 2. Molecular Features, Pre-Cursor Lesions and Carcinogenesis

There are generally considered to be two groups of epithelial ovarian cancer. Type I cancer comprises low-grade serous, low-grade endometrioid, clear cell and mucinous carcinomas. The behavior of these cancers is typically slower-growing, and they often present with tumor confined to the ovary, rather than disseminated disease. Type I cancers are often characterized by specific somatic mutations, including *KRAS*, *BRAF*, *ERBB2*, *CTNNB1*, *PTEN*, *PIK3CA*, *ARID1A*, and *PPP2R1A*, which target cell signaling pathways. Type I tumors rarely harbor *TP53* mutations as opposed to Tyle II tumors. These cancers appear to develop in a stepwise fashion from precursor lesions, such as borderline tumors and endometriosis.

Type II cancers include high-grade serous, high-grade endometrioid, malignant mixed mesodermal tumors (carcinosarcomas), and undifferentiated carcinomas. They are aggressive, present in advanced stage, and have a very high frequency of *TP53* mutations (>95%). Serous tubal intraepithelial carcinomas (STIC lesions) are now known to be very common precursor lesions for high-grade serous carcinomas and are also *TP53* mutated. STIC lesions spread from the open fimbriated ends of the fallopian tubes throughout the peritoneal cavity [8,9,10]. A large group of studies included patients with and without known genetic risk and confirmed that STICs and early invasive tubal carcinomas occurred in both groups. STIC lesions have also been identified in women without ovarian cancer who have their ovaries and tubes removed for other indications. The presence of identical TP53 mutations in STICs and concomitant ovarian high-grade serous cancers supports a clonal relationship between the two.

Identifying a known precursor lesion is an important step as scientists and clinicians attempt to detect ovarian cancer at earlier stages. Ovarian cancer development is a multistage process, the details of which are still being elucidated. The identification and characterization of oncogenes has provided the structural link between the carcinogen/promoter model of malignancy and the biochemical pathways that first become altered as cells become cancerous. Researchers have shown that malignant cells must have the ability to generate their own mitogenic signals, resist growth-inhibitory controls, evade apoptosis and to proliferate without limits while acquiring vasculature [11]. Additionally, the process of STIC lesions undergoing malignant transformation may produce changes in cellular metabolism. The above pathways put into context and offer opportunities to explore different approaches to the prevention and early detection of ovarian cancer.

## 3. Traditional and Novel Detection Methods

### 3.1. Circulating Biomarkers and Cytology

#### 3.1.1. Protein Biomarkers

Currently, Carbohydrate Antigen 125 (CA-125) and Human Epididymis protein 4 (HE4) are the only two markers that have been approved by the Federal Drug Administration (FDA) for monitoring treatment response and detecting disease recurrence in ovarian cancer patients. CA-125 is a mucin-type glycoprotein (MUC16) that is elevated in the serum of 83% of patients with ovarian cancer, but only in 50–60% of patients with stage I disease. Overexpression in other cancers, and benign diseases of the ovaries and reproductive tract (e.g., endometriosis), menstruation and pregnancy, give the test a lower specificity [12]. Due to low reliability (70% sensitivity and 87% specificity), CA-125 is not recommended for population screening [13].

Human epididymis protein (HE4) has been developed as a biomarker to improve the early detection of ovarian cancer. It is a glycoprotein belonging to the larger protein family called “WAP” for whey acidic proteins. It is secreted by epithelial ovarian cancer cells. HE4 levels are not elevated in endometriosis, which improves its specificity [14]. HE4 has proven the most useful in triaging patients with known pelvic masses to cancer specialists when indicated. Its sensitivity for detecting ovarian cancer is 65–83%, specificity is 78–99% [15].

Recently, OVERA^®^ was approved as referral or triage test for patients presenting with any type of ovarian mass, whether it be symptomatic or asymptomatic. The test is a combination of CA-125, HE4, apolipoprotein A-1, follicle-stimulating hormone (FSH), and transferrin. Previous studies have shown a relatively high sensitivity (91%) and a lower specificity (69%) limiting its clinical usefulness with a false positive rate similar to other serum markers [14].

Other blood biomarkers have been evaluated for their ability to detect early-stage ovarian cancer. Many have been tested in combination with CA-125. HE4 and CA-125, when combined, have a sensitivity of 76.4% and specificity of 95% for predicting if a pelvic mass is cancerous [16]. Other biomarkers of interest were identified from a large cohort of ovarian cancer screening studies, and include transthyretin, CA72.4, and CA15.3 [17]. Transthyretin is a plasma protein synthesized by the liver and plays a role in cellular metabolism. CA72.4 and CA15.3 are glycoproteins most commonly associated with gastrointestinal and breast cancers, respectively. These biomarkers were tested in 810 invasive epithelial ovarian cancer cases and controls from the European Prospective Investigation into Cancer and Nutrition (EPIC) study. Receiver operator performance was highest for CA-125 (0.92), followed by HE4 (0.84), CA72.4 (0.77), and CA15.3 (0.73) [18].

#### 3.1.2. Autoantibodies

Using proteomics, it is possible not only to identify new protein biomarkers, but also to find human immunoglobulin bound proteins and detect autoantibodies. Autoantibodies target tumor-associated antigens. Small volumes of cancer may not release adequate amounts of antigen to elevate serum protein levels, but could induce a human immune response and present an opportunity for identification of disease before widespread metastases or large primary tumors are present [19]. For example, the tumor suppressor gene *TP53* is mutated almost universally in high-grade serous ovarian cancers in addition to precursor STIC lesions [20]. TP53 autoantibody levels are increased in more than 20% of pre-treatment sera from patients with both early- and late-stage ovarian cancer. Previous studies showed that about 15% of ovarian cancer patients can be detected measuring autoantibodies against wild-type TP53 in sera [21]. Several other candidate autoantibodies have been identified, including homeobox gene A7 (HOXA7) interleukin 8 (IL-8), TRIM21, and NY-ESO01 [22,23,24], all of which are under investigation.

#### 3.1.3. MicroRNAs

MicroRNAs (miRNAs) are short (~22 nucleotides), single-stranded non-coding RNAs that play a key role in regulating gene expression at the post-transcriptional level [25]. These molecules are found circulating in various body fluids, where they remain stable by associating with proteins or being enclosed in exosomes, microvesicles, or apoptotic bodies. Because their expression patterns shift in response to pathological conditions (like cancer), circulating miRNAs are being explored as promising biomarkers for cancer detection [26,27]. In ovarian cancer, miRNA expression is often dysregulated, which can be useful in distinguishing malignant cells from normal ovarian tissue [28].

**Figure 1:**
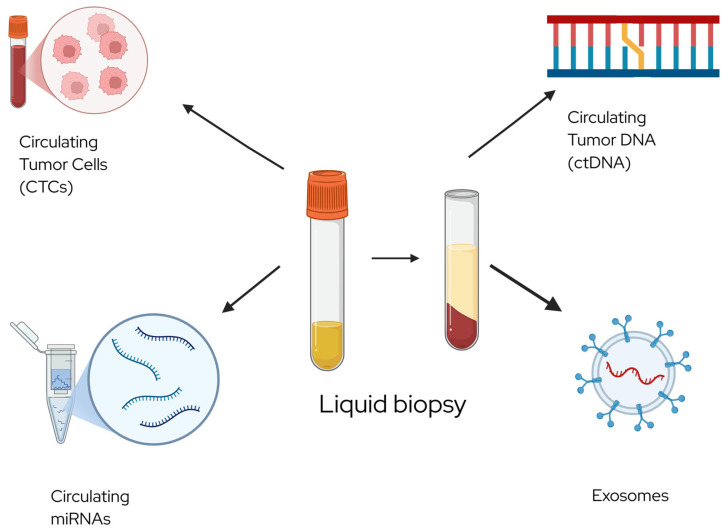
Examples of output from liquid biopsy. Created in BioRender. Barroilhet, L. (2025) https://BioRender.com/telkoq3 (accessed on 6 October 2025).

Several serum miRNAs (Figure 1), including miR-182 and members of the miR-200 family (miR-200a, miR-200b, miR-200c), are elevated in patients with serous epithelial ovarian cancer (EOC). Notably, the combination of miR-200b and miR-200c demonstrated good diagnostic performance, with an AUC of 0.784 for distinguishing serous EOC from healthy controls [26]. Another combination—miR-205 and let-7f—showed even higher diagnostic accuracy (AUC = 0.831, 95% CI: 0.772–0.880), with 62.4% sensitivity and 92.9% specificity, particularly effective in detecting stage I disease [29]. Additionally, an eight-miRNA panel was able to differentiate early-stage EOC from benign ovarian tumors with 86% sensitivity and 83% specificity [30,31]. Ongoing prospective clinical trials, such as NCT05146505, are currently evaluating the utility of miRNAs for early ovarian cancer detection.

#### 3.1.4. Urine Testing

The potential of urine as a source of ovarian cancer biomarkers is the subject of ongoing research [32]. Eosinophil protein X, a glycosylated form of eosinophil-derived neurotoxin and carboxylic acid-terminated fragments of osteopontin [33] have both been detected in the urine of ovarian cancer patients. Interestingly, eosinophil protein X has been shown to have antitumor and antiangiogenic activity via induction of apoptosis of endothelial cells and may also have therapeutic potential. Osteopontin is correlated with systemic inflammation. Potential urine biomarkers also include HE4 [34] and HMGA1 [35].

#### 3.1.5. Circulating Tumor DNA

Circulating tumor DNA (ctDNA) refers to the fraction of cell-free DNA in the bloodstream that carries tumor-specific somatic mutations [36]. Assessing ctDNA in serum offers a promising, noninvasive approach for cancer detection and disease monitoring [37]. Advances in assay technologies have significantly enhanced sensitivity, enabling detection of cancers at early stages when ctDNA levels are very low. In a small cohort of seven ovarian cancer patients, Bettegowda et al. reported ctDNA detection in all cases (100%) [38]. Targeted deep sequencing has further improved detection, identifying mutations at allelic frequencies as low as 2% with greater than 97% sensitivity and specificity [39].

A novel multi-analyte blood test, CancerSEEK, combines analysis of eight protein biomarkers—including CA-125—with mutation screening across 1933 genomic sites, encompassing single-base substitutions, insertions, and deletions [40]. In a case–control study spanning eight cancer types, CancerSEEK demonstrated its highest accuracy in ovarian cancer, achieving 98% sensitivity and over 99% specificity, with only seven false positives among 812 cancer-free individuals. However, most participants had advanced-stage disease, which could have been detected using CA-125 alone. Further validation in early-stage ovarian cancer patients remains essential [40].

#### 3.1.6. DNA Methylation

Aberrant DNA methylation, particularly in promoter regions, is recognized as an early molecular event in the development of cancer [31]. This epigenetic alteration can disrupt normal gene regulation, leading to silencing of tumor suppressor genes or activation of oncogenes [41]. In a study involving 250 patients with epithelial ovarian cancer (EOC) undergoing chemotherapy, along with a larger cohort from an ultrasound-based screening trial, a panel of three serum DNA methylation markers was evaluated. These markers successfully identified more than half of ovarian cancer cases in blood samples collected up to two years before clinical diagnosis [42]. While similar methylation-based approaches have been effectively translated into clinical diagnostics for colorectal cancer, the diverse histological subtypes of ovarian cancer present a significant obstacle to creating a biomarker panel that is both sensitive and specific [43].

#### 3.1.7. Microbiome

The human body is colonized by diverse communities of microorganisms that reside within the mucosal surfaces, known as the microbiome. The microbiome has many necessary and important functions, including nutrient absorption, metabolism of hormones and vitamins, immune system modulation, and protection against pathogenic infections. Contemporary next-generation sequencing technologies have advanced our understanding that distinct bacterial populations also inhabit the uterus, fallopian tubes, and peritoneum [36,44].

In the majority of women of reproductive age, the microbiota of vagina and cervix microenvironment is dominated by Lactobacillus species. By contrast, the uterus, ovaries and fallopian tubes are sterile in healthy individual. Dysbiosis, or alterations to the bacterial communities and their diversity, within the microbiome has been associated with many diseases of the female reproductive tract, such as recurrent urinary tract infections, endometriosis, infertility, pre-invasive disease, and gynecologic cancers. When dysbiosis occurs, altered immune and metabolic signaling can cause chronic inflammation, epithelial barrier breach, changes in cellular proliferation and apoptosis, genome instability, angiogenesis and metabolic dysregulation. These pathophysiological changes could be a part of the complicated process of carcinogenesis in gynecologic organs [45]. Certain microbes can elicit DNA damage and apoptosis directly via the production of toxic metabolites or indirectly via reactive oxygen species. Furthermore, bacteria associated with malignant transformation, such as *Salmonella enterica* serovar *typhi*, *Helicobacter pylori*, and *Fusobacteria* spp., have been shown to upregulate oncogenic pathways, including B-catenin signaling, and increase the production of pro-inflammatory cytokines [46]. Differences between the microbiome in benign and malignant disease processes are being studied (NCT 03388996).

#### 3.1.8. Fallopian Tube Cytology and Tumor DNA Detection in Pap Smears

Recognition that many ovarian cancers arise in the fallopian tube has resulted in the development of fallopian tube tissue sampling devices. More recently, in a small feasibility study, Lum et al. attempted cytologic sampling of the fallopian tubes using hysteroscopic brush cytology without visualization [47]. While they were able to perform cellular collection laparoscopically, hysteroscopic methods were less successful [48]. Others have verified cytologic sampling from excised tubes [49], but again, this has not been duplicated consistently using a less invasive hysteroscopic approach. Several groups have started to characterize the cytology of the fallopian tube as well as endometrial cytology for early detection of ovarian cancer, although further analysis and standardization is needed [50,51].

Liquid-based cervical Pap smear testing, which combines cytological evaluation with DNA analysis, has recently been explored as a potential screening method for cancers beyond the cervix [52]. In a study by Kinde et al., DNA mutations were detected in liquid Pap smear samples from 100% of patients with endometrial cancer and 41% of those with ovarian cancer [53]. Additionally, in a small cohort of patients with advanced high-grade serous ovarian cancer, TP53 mutations were identified in three out of five individuals who had intact fallopian tubes and used a tampon prior to surgery. In contrast, none of the three patients with prior tubal ligation showed detectable mutations [20].

### 3.2. Imaging

#### 3.2.1. Ultrasound with Contrast

Ultrasound is a safe, widely available, and relatively inexpensive imaging modality commonly used to visualize the female pelvic organs. However, even for screening high-risk populations for ovarian cancer, its utility has been limited. The introduction of molecularly targeted contrast microbubbles that can bind to specific molecules expressed in ovarian cancer has been the subject of recent research studies to improve early detection and potentially drug delivery [54,55,56].

Contrast microbubbles are micron-sized, intravascular contrast agents consisting of a gaseous core contained by a shell. The introduction of molecularly targeted contrast microbubbles that can bind to certain molecules expressed in cancer has made ultrasound a molecular imaging modality that allows improved detection, characterization, and monitoring. By functionalizing the shell with binding ligands to certain molecules, microbubble-enhanced ultrasound can visualize molecules such as kinase insert domain receptor (KDR), one of the key regulators of angiogenesis preferentially expressed in various cancers, including ovarian cancer [55].

Twenty-four women (age 48 to 79 years) with focal ovarian lesions were injected intravenously with MBKDR (0.03 to 0.08 mL/kg of body weight) ultrasound of the ovaries was performed starting 5 min after injection. Persistent focal MBKDR binding was assessed. Patients underwent surgical resection of the target lesions, and tissues were stained for CD31 and KDR by immunohistochemistry. Overall, the safety profile of MBKDR was good, with infrequent mild or moderate self-resolving adverse events [55]. Although microbubble enhancement holds promise for improving specificity, it may not improve detection of early-stage disease. In the case of precancerous lesions within the fallopian tubes, microbubbles may not add any diagnostic advantage.

#### 3.2.2. Magnetic Resonance Imaging

Both magnetic resonance imaging (MRI) and magnetic relaxometry (MRX) can detect and locate magnetic nanoparticles without ionizing radiation. MRX is a highly sensitive technique that detects and quantifies magnetic nanoparticles in biological tissues by measuring their decay after removal of an external magnetic field. MRX is being studied for the early detection of many cancers, but is not ready for routine adaption into clinical practice [57,58]. For ovarian cancer, it holds the most promise in distinguishing benign endometriosis lesions from endometriosis-associated ovarian cancer [59,60].

## 4. Novel Prevention Methods

As discussed above, early detection methods for ovarian cancer are evolving, with no consistent recommendations for screening even high-risk patients. This informs the landscape (and underscores the importance) of ovarian cancer prevention [61].

### 4.1. Salpingectomy

Large clinical screening trials using transvaginal ultrasound and serum CA-125 have not demonstrated a reduction in EOC specific mortality. Accordingly, development of sensitive early detection methods that achieve high specificity represents a critical unmet need. Opportunistic salpingectomy performed as an alternative to tubal ligation at the time of hysterectomy can prevent cancers originating in the fallopian tubes [61]. Opportunistic salpingectomy at the time of cesarean delivery has replaced tubal ligation as the most common type of sterilization [62]. Postpartum opportunistic salpingectomy after vaginal delivery has also been shown to be a cost effective and safe option [63,64].

Removal of the ovaries and fallopian tubes is an effective approach to reducing ovarian cancer risk and is routinely recommended in high-risk populations. While this approach is very effective (>95% risk reduction), it often comes with undesirable side effects, such as a truncated fertility window and the negative effects of early menopause [65]. A proposed approach in high-risk women who are premenopausal is tubal removal (salpingectomy) with delayed oophorectomy closer to the age of natural menopause. Salpingectomy does not affect ovarian reserve in the short term and provides effective non-hormonal birth control. Concerns with salpingectomy for ovarian cancer reduction include the possibility of residual tubal tissue remaining on the ovarian surface. This concern was validated when tubes and ovaries were removed separately during surgery and examined histologically as two separate specimens; residual fimbrial tissue was found on the ovarian surface in 16% of cases [66]. This suggests that salpingectomy may not prevent all ovarian cancers of tubal origin. Ongoing, prospective, observational studies are following up participants who choose among different surgical prevention options. These studies include TUBA-WISP-II [67] (international), SOROCk (US) [68] and PROTECTOR (UK) [69].

Opportunistic salpingectomy is also being studied as a risk reduction technique in the general population. In this model, average-risk patients who are having abdominal surgery for another indication have their fallopian tubes removed at the same time. Retrospective population-based data on bilateral salpingectomy with ovarian conservation from Sweden and Denmark suggest that it is associated with a 42% and 65% ovarian cancer risk reduction, respectively [70,71]. Both studies compared salpingectomy with no surgery as opposed to hysterectomy without salpingectomy. We are awaiting mature survival data from a retrospective cohort studied entitled Hysterectomy and Opportunistic Salpingectomy [72]. Currently there is insufficient evidence to accurately estimate the magnitude of EOC risk reduction with opportunistic salpingectomy in average risk patients or salpingectomy alone in high-risk populations.

### 4.2. Chemoprevention

Combined oral contraceptives (COCs) have long been used to reduce ovarian cancer risk, although their use is more controversial in BRCA positive and other high-risk populations, as COC use has been linked to an increased risk of breast cancer [73]. A recently published meta-analysis found eight cohort studies that investigated oral contraceptive use in relation to ovarian cancer rate. Compared with non-users, all hormonal contraceptive users had a 36% lower rate ratio of ovarian cancer (0.65, 95% CI 0.60, 0.68) [74] without an increase in breast cancer risk. From a prevention standpoint, combined (estrogen/progesterone) oral contraceptives are the most effective non-surgical method available. Continuous COC used for more than 5 years can reduce the risk of ovarian cancer by >50% [75].

A recent meta-analysis based on retrospective data found that any intrauterine device (IUD) use, a common contraceptive option, was associated with a 32% reduction in the incidence of ovarian cancer [76].

Many analgesic drugs have been studied for the prevention of ovarian cancer, with mixed results. Meta-analyses and pooled analyses of cohort and case–control studies have found that aspirin may reduce ovarian cancer risk by 10–20%, particularly when used frequently (i.e., daily or almost daily) [77,78,79,80]. Aspirin is now being used as a cancer preventing agent (600 mg/day for at least two years) for Lynch syndrome patients, based on its ability to reduce the risk of colorectal, ovarian and endometrial cancers [81]. Results surrounding NSAID and acetaminophen use as it relates to ovarian cancer risk reduction are inconsistent [82,83,84].

Natural compounds with antiangiogenic properties, such as curcumin, resveratrol, and silibinin have shown promising preclinical effects in cancer prevention [85,86,87,88,89] as have retinoids [90]. Small molecular inhibitors that block oxidative phosphorylation and may prevent early changes in cellular metabolism related to glycolysis are also being studied [91].

## 5. Conclusions and Future Directions

In conclusion, multiple innovative methods for early detection and prevention are under investigation. The current state focuses on identification of high-risk patients and early adoption of chemoprevention or surgical tactics to reduce risk. Although successful early detection of ovarian cancer would undoubtedly save lives, no imaging, plasma or urine study is currently FDA-approved for this purpose. Stepwise or multi-modality screening may enhance sensitivity and specificity. Population-based screening is not currently recommended, as no screening test has been able to demonstrate reduced mortality from ovarian cancer, even when implemented in a high-risk population.

## Abbreviations

The following abbreviations are used in this manuscript:

CA-125	Carbohydrate Antigen 125
DNA	Deoxyribonucleic acid
EOC	Epithelial ovarian cancer
HE4	Human epididymis protein 4
HGSOC	High-grade serous ovarian cancer
HMGA	High mobility group A
IUD	Intrauterine device
MRA	Magnetic relaxometry
MRI	Magnetic resonance imaging
NSAID	Non-steroidal anti-inflammatory drugs
STIC	Serous tubal intraepithelial carcinoma

## References

[1] Siegel R.L., Miller K.D., Fuchs H.E., Jemal A. (2021). Cancer Statistics, 2021. CA Cancer J. Clin..

[2] Goff B.A., Mandel L., Muntz H.G., Melancon C.H. (2000). Ovarian carcinoma diagnosis. Cancer.

[3] Berek J.S., Renz M., Kehoe S., Kumar L., Friedlander M. (2021). Cancer of the ovary, fallopian tube, and peritoneum: 2021 update. Int. J. Gynaecol. Obstet..

[4] Torre L.A., Trabert B., DeSantis C.E., Miller K.D., Samimi G., Runowicz C.D., Gaudet M.M., Jemal A., Siegel R.L. (2018). Ovarian cancer statistics, 2018. CA Cancer J. Clin..

[5] Kotsopoulos J., Hathaway C.A., Narod S.A., Teras L.R., Patel A.V., Hu C., Yadav S., Couch F.J., Tworoger S.S. (2023). Germline Mutations in 12 Genes and Risk of Ovarian Cancer in Three Population-Based Cohorts. Cancer Epidemiol. Biomark. Prev..

[6] Konstantinopoulos P.A., Norquist B., Lacchetti C., Armstrong D., Grisham R.N., Goodfellow P.J., Kohn E.C., Levine D.A., Liu J.F., Lu K.H. (2020). Germline and Somatic Tumor Testing in Epithelial Ovarian Cancer: ASCO Guideline. J. Clin. Oncol..

[7] Webb P.M., Jordan S.J. (2017). Epidemiology of epithelial ovarian cancer. Best Pract. Res. Clin. Obstet. Gynaecol..

[8] Roh M.H., Kindelberger D., Crum C.P. (2009). Serous tubal intraepithelial carcinoma and the dominant ovarian mass: Clues to serous tumor origin?. Am. J. Surg. Pathol..

[9] Crum C.P., Drapkin R., Miron A., Ince T.A., Muto M., Kindelberger D.W., Lee Y. (2007). The distal fallopian tube: A new model for pelvic serous carcinogenesis. Curr. Opin. Obstet. Gynecol..

[10] Gockley A.A., Elias K.M. (2018). Fallopian tube tumorigenesis and clinical implications for ovarian cancer risk-reduction. Cancer Treat. Rev..

[11] Hahn W.C., Weinberg R.A. (2002). Rules for making human tumor cells. N. Engl. J. Med..

[12] Bast R.C., Xu F.J., Yu Y.H., Barnhill S., Zhang Z., Mills G.B. (1998). CA 125: The past and the future. Int. J. Biol. Markers.

[13] Moss E.L., Hollingworth J., Reynolds T.M. (2005). The role of CA125 in clinical practice. J. Clin. Pathol..

[14] Dochez V., Caillon H., Vaucel E., Dimet J., Winer N., Ducarme G. (2019). Biomarkers and algorithms for diagnosis of ovarian cancer: CA125, HE4, RMI and ROMA, a review. J. Ovarian Res..

[15] Caruso G., Weroha S.J., Cliby W. (2025). Ovarian Cancer: A Review. JAMA.

[16] Moore R.G., Brown A.K., Miller M.C., Skates S., Allard W.J., Verch T., Steinhoff M., Messerlian G., DiSilvestro P., Granai C.O. (2008). The use of multiple novel tumor biomarkers for the detection of ovarian carcinoma in patients with a pelvic mass. Gynecol. Oncol..

[17] Cramer D.W., Bast R.C., Berg C.D., Diamandis E.P., Godwin A.K., Hartge P., Lokshin A.E., Lu K.H., McIntosh M.W., Mor G. (2011). Ovarian cancer biomarker performance in prostate, lung, colorectal, and ovarian cancer screening trial specimens. Cancer Prev. Res..

[18] Terry K.L., Schock H., Fortner R.T., Hüsing A., Fichorova R.N., Yamamoto H.S., Vitonis A.F., Johnson T., Overvad K., Tjønneland A. (2016). A Prospective Evaluation of Early Detection Biomarkers for Ovarian Cancer in the European EPIC Cohort. Clin. Cancer Res..

[19] Macdonald I.K., Parsy-Kowalska C.B., Chapman C.J. (2017). Autoantibodies: Opportunities for Early Cancer Detection. Trends Cancer.

[20] Erickson B.K., Kinde I., Dobbin Z.C., Wang Y., Martin J.Y., Alvarez R.D., Conner M.G., Huh W.K., Roden R.B.S., Kinzler K.W. (2014). Detection of somatic TP53 mutations in tampons of patients with high-grade serous ovarian cancer. Obstet. Gynecol..

[21] Yang W.L., Gentry-Maharaj A., Simmons A., Ryan A., Fourkala E.O., Lu Z., Baggerly K.A., Zhao Y., Lu K.H., Bowtell D. (2017). Elevation of TP53 Autoantibody Before CA125 in Preclinical Invasive Epithelial Ovarian Cancer. Clin. Cancer Res..

[22] Hurley L.C., Levin N.K., Chatterjee M., Coles J., Muszkat S., Howarth Z., Dyson G., Tainsky M.A. (2020). Evaluation of paraneoplastic antigens reveals TRIM21 autoantibodies as biomarker for early detection of ovarian cancer in combination with autoantibodies to NY-ESO-1 and TP53. Cancer Biomark..

[23] Fortner R.T., Damms-Machado A., Kaaks R. (2017). Systematic review: Tumor-associated antigen autoantibodies and ovarian cancer early detection. Gynecol. Oncol..

[24] Lokshin A.E., Winans M., Landsittel D., Marrangoni A.M., Velikokhatnaya L., Modugno F., Nolen B.M., Gorelik E. (2006). Circulating IL-8 and anti-IL-8 autoantibody in patients with ovarian cancer. Gynecol. Oncol..

[25] Saliminejad K., Khorram Khorshid H.R., Soleymani Fard S., Ghaffari S.H. (2019). An overview of microRNAs: Biology, functions, therapeutics, and analysis methods. J. Cell. Physiol..

[26] Kan C.W., Hahn M.A., Gard G.B., Maidens J., Huh J.Y., Marsh D.J., Howell V.M. (2012). Elevated levels of circulating microRNA-200 family members correlate with serous epithelial ovarian cancer. BMC Cancer.

[27] Ghafouri-Fard S., Shoorei H., Taheri M. (2020). miRNA profile in ovarian cancer. Exp. Mol. Pathol..

[28] Yokoi A., Yoshioka Y., Hirakawa A., Yamamoto Y., Ishikawa M., Ikeda S.I., Kato T., Niimi K., Kajiyama H., Kikkawa F. (2017). A combination of circulating miRNAs for the early detection of ovarian cancer. Oncotarget.

[29] Zheng H., Zhang L., Zhao Y., Yang D., Song F., Wen Y., Hao Q., Hu Z., Zhang W., Chen K. (2013). Plasma miRNAs as diagnostic and prognostic biomarkers for ovarian cancer. PLoS ONE.

[30] Feng S., Pan W., Jin Y., Zheng J. (2014). MiR-25 promotes ovarian cancer proliferation and motility by targeting LATS2. Tumour Biol..

[31] Wang S., Wu W., Claret F.X. (2017). Mutual regulation of microRNAs and DNA methylation in human cancers. Epigenetics.

[32] Chambers A.F., Vanderhyden B.C. (2006). Ovarian cancer biomarkers in urine. Clin. Cancer Res..

[33] Rani S., Sehgal A., Kaur J., Pandher D.K., Punia R.S. (2022). Osteopontin as a Tumor Marker in Ovarian Cancer. J. Midlife Health.

[34] Jia M.M., Deng J., Cheng X.L., Yan Z., Li Q.C., Xing Y.Y., Fan D.M., Tian X.Y. (2017). Diagnostic accuracy of urine HE4 in patients with ovarian cancer: A meta-analysis. Oncotarget.

[35] Zhou J., Xie M., He H., Shi Y., Luo B., Gong G., Li J., Wang J., Wu X., Wen J. (2015). Increases urinary HMGA1 in serous epithelial ovarian cancer patients. Cancer Biomark..

[36] Cheng F., Su L., Qian C. (2016). Circulating tumor DNA: A promising biomarker in the liquid biopsy of cancer. Oncotarget.

[37] Pantel K., Alix-Panabières C. (2019). Liquid biopsy and minimal residual disease—Latest advances and implications for cure. Nat. Rev. Clin. Oncol..

[38] Bettegowda C., Sausen M., Leary R.J., Kinde I., Wang Y., Agrawal N., Bartlett B.R., Wang H., Luber B., Alani R.M. (2014). Detection of circulating tumor DNA in early- and late-stage human malignancies. Sci. Transl. Med..

[39] Forshew T., Murtaza M., Parkinson C., Gale D., Tsui D.W., Kaper F., Dawson S.J., Piskorz A.M., Jimenez-Linan M., Bentley D. (2012). Noninvasive identification and monitoring of cancer mutations by targeted deep sequencing of plasma DNA. Sci. Transl. Med..

[40] Cohen J.D., Li L., Wang Y., Thoburn C., Afsari B., Danilova L., Douville C., Javed A.A., Wong F., Mattox A. (2018). Detection and localization of surgically resectable cancers with a multi-analyte blood test. Science.

[41] Gao Y., Zhou N., Liu J. (2024). Ovarian Cancer Diagnosis and Prognosis Based on Cell-Free DNA Methylation. Cancer Control.

[42] Widschwendter M., Zikan M., Wahl B., Lempiäinen H., Paprotka T., Evans I., Jones A., Ghazali S., Reisel D., Eichner J. (2017). The potential of circulating tumor DNA methylation analysis for the early detection and management of ovarian cancer. Genome Med..

[43] Papakonstantinou E., Androutsopoulos G., Logotheti S., Adonakis G., Maroulis I., Tzelepi V. (2021). DNA Methylation in Epithelial Ovarian Cancer: Current Data and Future Perspectives. Curr. Mol. Pharmacol..

[44] Łaniewski P., Ilhan Z.E., Herbst-Kralovetz M.M. (2020). The microbiome and gynaecological cancer development, prevention and therapy. Nat. Rev. Urol..

[45] Siddiqui R., Makhlouf Z., Alharbi A.M., Alfahemi H., Khan N.A. (2022). The Gut Microbiome and Female Health. Biology.

[46] Sipos A., Ujlaki G., Mikó E., Maka E., Szabó J., Uray K., Krasznai Z., Bai P. (2021). The role of the microbiome in ovarian cancer: Mechanistic insights into oncobiosis and to bacterial metabolite signaling. Mol. Med..

[47] Lum D., Guido R., Rodriguez E., Lee T., Mansuria S., D’Ambrosio L., Austin R.M. (2014). Brush cytology of the fallopian tube and implications in ovarian cancer screening. J. Minim. Invasive Gynecol..

[48] Maritschnegg E., Wang Y., Pecha N., Horvat R., Van Nieuwenhuysen E., Vergote I., Heitz F., Sehouli J., Kinde I., Diaz L.A. (2015). Lavage of the Uterine Cavity for Molecular Detection of Müllerian Duct Carcinomas: A Proof-of-Concept Study. J. Clin. Oncol..

[49] Chen H., Klein R., Arnold S., Chambers S., Zheng W. (2016). Cytologic studies of the fallopian tube in patients undergoing salpingo-oophorectomy. Cancer Cell Int..

[50] Otsuka I., Kameda S., Hoshi K. (2013). Early detection of ovarian and fallopian tube cancer by examination of cytological samples from the endometrial cavity. Br. J. Cancer.

[51] Rodriguez E.F., Lum D., Guido R., Austin R.M. (2013). Cytologic findings in experimental in vivo fallopian tube brush specimens. Acta Cytol..

[52] Wang Y., Li L., Douville C., Cohen J.D., Yen T.T., Kinde I., Sundfelt K., Kjær S.K., Hruban R.H., Shih I.M. (2018). Evaluation of liquid from the Papanicolaou test and other liquid biopsies for the detection of endometrial and ovarian cancers. Sci. Transl. Med..

[53] Kinde I., Bettegowda C., Wang Y., Wu J., Agrawal N., Shih Ie M., Kurman R., Dao F., Levine D.A., Giuntoli R. (2013). Evaluation of DNA from the Papanicolaou test to detect ovarian and endometrial cancers. Sci. Transl. Med..

[54] Ba S., Yu M. (2022). Ultrasound-stimulated microbubbles enhances radiosensitivity of ovarian cancer. Acta Radiol..

[55] Willmann J.K., Bonomo L., Testa A.C., Rinaldi P., Rindi G., Valluru K.S., Petrone G., Martini M., Lutz A.M., Gambhir S.S. (2017). Ultrasound Molecular Imaging with BR55 in Patients with Breast and Ovarian Lesions: First-in-Human Results. J. Clin. Oncol..

[56] Luo T., Sun J., Zhu S., He J., Hao L., Xiao L., Zhu Y., Wang Q., Pan X., Wang Z. (2017). Ultrasound-mediated destruction of oxygen and paclitaxel loaded dual-targeting microbubbles for intraperitoneal treatment of ovarian cancer xenografts. Cancer Lett..

[57] Nebgen D.R., Lu K.H., Bast R.C. (2019). Novel Approaches to Ovarian Cancer Screening. Curr. Oncol. Rep..

[58] Vandsburger M.H., Radoul M., Addadi Y., Mpofu S., Cohen B., Eilam R., Neeman M. (2013). Ovarian carcinoma: Quantitative biexponential MR imaging relaxometry reveals the dynamic recruitment of ferritin-expressing fibroblasts to the angiogenic rim of tumors. Radiology.

[59] Matsubara S., Kawahara N., Horie A., Murakami R., Horikawa N., Sumida D., Wada T., Maehana T., Yamawaki A., Ichikawa M. (2019). Magnetic resonance relaxometry improves the accuracy of conventional MRI in the diagnosis of endometriosis-associated ovarian cancer: A case report. Mol. Clin. Oncol..

[60] Kawahara N., Kobayashi H., Maehana T., Iwai K., Yamada Y., Kawaguchi R., Takahama J., Marugami N., Nishi H., Sakai Y. (2024). MR Relaxometry for Discriminating Malignant Ovarian Cystic Tumors: A Prospective Multicenter Cohort Study. Diagnostics.

[61] Lawton F.G., Pavlik E.J. (2022). Perspectives on Ovarian Cancer 1809 to 2022 and Beyond. Diagnostics.

[62] Rufin K.G.A., do Valle H.A., McAlpine J.N., Elwood C., Hanley G.E. (2024). Complications after opportunistic salpingectomy compared with tubal ligation at cesarean section: A retrospective cohort study. Fertil. Steril..

[63] Wagar M.K., Forlines G.L., Moellman N., Carlson A., Matthews M., Williams M. (2023). Postpartum Opportunistic Salpingectomy Compared with Bilateral Tubal Ligation After Vaginal Delivery for Ovarian Cancer Risk Reduction: A Cost-Effectiveness Analysis. Obstet. Gynecol..

[64] Wagar M.K., Godecker A., Landeros M.V., Williams M. (2021). Postpartum Salpingectomy Compared with Standard Tubal Ligation After Vaginal Delivery. Obstet. Gynecol..

[65] Collins E., Strandell A., Granåsen G., Idahl A. (2019). Menopausal symptoms and surgical complications after opportunistic bilateral salpingectomy, a register-based cohort study. Am. J. Obstet. Gynecol..

[66] Gan C., Chenoy R., Chandrasekaran D., Brockbank E., Hollingworth A., Vimplis S., Lawrence A.C., Jeyarajah A.R., Oram D., Deo N. (2017). Persistence of fimbrial tissue on the ovarian surface after salpingectomy. Am. J. Obstet. Gynecol..

[67] Steenbeek M.P., van Bommel M.H.D., intHout J., Peterson C.B., Simons M., Roes K.C.B., Kets M., Norquist B.M., Swisher E.M., Hermens R. (2023). TUBectomy with delayed oophorectomy as an alternative to risk-reducing salpingo-oophorectomy in high-risk women to assess the safety of prevention: The TUBA-WISP II study protocol. Int. J. Gynecol. Cancer.

[68] Huh W.K., Pugh S.L., Walker J.L., Pennington K., Jewell E.L., Havrilesky L.J., Carter J., Muller C., Drapkin R., Lankes H.A. (2022). NRG-CC008: A nonrandomized prospective clinical trial comparing the non-inferiority of salpingectomy to salpingo-oophorectomy to reduce the risk of ovarian cancer among BRCA1 carriers [SOROCk]. Am. Soc. Clin. Oncol..

[69] Gaba F., Robbani S., Singh N., McCluggage W.G., Wilkinson N., Ganesan R., Bryson G., Rowlands G., Tyson C., Arora R. (2021). Preventing Ovarian Cancer through early Excision of Tubes and late Ovarian Removal (PROTECTOR): Protocol for a prospective non-randomised multi-center trial. Int. J. Gynecol. Cancer.

[70] Falconer H., Yin L., Grönberg H., Altman D. (2015). Ovarian cancer risk after salpingectomy: A nationwide population-based study. J. Natl. Cancer Inst..

[71] Guldberg R., Wehberg S., Skovlund C.W., Mogensen O., Lidegaard O. (2013). Salpingectomy as standard at hysterectomy? A Danish cohort study, 1977–2010. BMJ Open.

[72] Idahl A., Darelius A., Sundfeldt K., Pålsson M., Strandell A. (2019). Hysterectomy and opportunistic salpingectomy (HOPPSA): Study protocol for a register-based randomized controlled trial. Trials.

[73] Heimdal K., Skovlund E., Møller P. (2002). Oral contraceptives and risk of familial breast cancer. Cancer Detect. Prev..

[74] Jahanfar S., Mortazavi J., Lapidow A., Cu C., Al Abosy J., Morris K., Becerra-Mateus J.C., Steinfeldt M., Maurer O., Bohang J. (2024). Assessing the impact of contraceptive use on reproductive cancer risk among women of reproductive age-a systematic review. Front. Glob. Womens Health.

[75] Beral V., Doll R., Hermon C., Peto R., Reeves G. (2008). Ovarian cancer and oral contraceptives: Collaborative reanalysis of data from 45 epidemiological studies including 23,257 women with ovarian cancer and 87,303 controls. Lancet.

[76] Wheeler L.J., Desanto K., Teal S.B., Sheeder J., Guntupalli S.R. (2019). Intrauterine Device Use and Ovarian Cancer Risk: A Systematic Review and Meta-analysis. Obstet. Gynecol..

[77] Zhang D., Bai B., Xi Y., Wang T., Zhao Y. (2016). Is aspirin use associated with a decreased risk of ovarian cancer? A systematic review and meta-analysis of observational studies with dose-response analysis. Gynecol. Oncol..

[78] Trabert B., Poole E.M., White E., Visvanathan K., Adami H.O., Anderson G.L., Brasky T.M., Brinton L.A., Fortner R.T., Gaudet M. (2019). Analgesic Use and Ovarian Cancer Risk: An Analysis in the Ovarian Cancer Cohort Consortium. J. Natl. Cancer Inst..

[79] Hurwitz L.M., Webb P.M., Jordan S.J., Doherty J.A., Harris H.R., Goodman M.T., Shvetsov Y.B., Modugno F., Moysich K.B., Schildkraut J.M. (2023). Association of Frequent Aspirin Use with Ovarian Cancer Risk According to Genetic Susceptibility. JAMA Netw. Open.

[80] Trabert B., Ness R.B., Lo-Ciganic W.H., Murphy M.A., Goode E.L., Poole E.M., Brinton L.A., Webb P.M., Nagle C.M., Jordan S.J. (2014). Aspirin, nonaspirin nonsteroidal anti-inflammatory drug, and acetaminophen use and risk of invasive epithelial ovarian cancer: A pooled analysis in the Ovarian Cancer Association Consortium. J. Natl. Cancer Inst..

[81] Burn J., Sheth H., Elliott F., Reed L., Macrae F., Mecklin J.P., Möslein G., McRonald F.E., Bertario L., Evans D.G. (2020). Cancer prevention with aspirin in hereditary colorectal cancer (Lynch syndrome), 10-year follow-up and registry-based 20-year data in the CAPP2 study: A double-blind, randomised, placebo-controlled trial. Lancet.

[82] Moysich K.B., Mettlin C., Piver M.S., Natarajan N., Menezes R.J., Swede H. (2001). Regular use of analgesic drugs and ovarian cancer risk. Cancer Epidemiol. Biomark. Prev..

[83] Meier C.R., Schmitz S., Jick H. (2002). Association between acetaminophen or nonsteroidal antiinflammatory drugs and risk of developing ovarian, breast, or colon cancer. Pharmacotherapy.

[84] Sasamoto N., Babic A., Vitonis A.F., Titus L., Cramer D.W., Trabert B., Tworoger S.S., Terry K.L. (2021). Common Analgesic Use for Menstrual Pain and Ovarian Cancer Risk. Cancer Prev. Res..

[85] Gor R., Ramachandran I., Ramalingam S. (2022). Targeting the Cancer Stem Cells in Endocrine Cancers with Phytochemicals. Curr. Top. Med. Chem..

[86] Casey S.C., Amedei A., Aquilano K., Azmi A.S., Benencia F., Bhakta D., Bilsland A.E., Boosani C.S., Chen S., Ciriolo M.R. (2015). Cancer prevention and therapy through the modulation of the tumor microenvironment. Semin. Cancer Biol..

[87] Milani A., Basirnejad M., Shahbazi S., Bolhassani A. (2017). Carotenoids: Biochemistry, pharmacology and treatment. Br. J. Pharmacol..

[88] Xu X.L., Deng S.L., Lian Z.X., Yu K. (2021). Resveratrol Targets a Variety of Oncogenic and Oncosuppressive Signaling for Ovarian Cancer Prevention and Treatment. Antioxidants.

[89] Cho H.J., Suh D.S., Moon S.H., Song Y.J., Yoon M.S., Park D.Y., Choi K.U., Kim Y.K., Kim K.H. (2013). Silibinin inhibits tumor growth through downregulation of extracellular signal-regulated kinase and Akt in vitro and in vivo in human ovarian cancer cells. J. Agric. Food Chem..

[90] Veronesi U., Decensi A. (2001). Retinoids for ovarian cancer prevention: Laboratory data set the stage for thoughtful clinical trials. J. Natl. Cancer Inst..

[91] Kapur A., Mehta P., Simmons A.D., Ericksen S.S., Mehta G., Palecek S.P., Felder M., Stenerson Z., Nayak A., Dominguez J.M.A. (2022). Atovaquone: An Inhibitor of Oxidative Phosphorylation as Studied in Gynecologic Cancers. Cancers.

